# Notes and News

**Published:** 1897-05-08

**Authors:** 


### Notes and News.

(Collected and Communicated.)

A grand special matinée in commemoration of the Diamond Jubilee will be given at the Tivoli on Monday, May 10th, in aid of the funds of Charing Cross Hospital.

The Special Appeal Fund Committee of Charing Cross Hospital have received £210 from the directors of the London Pavilion as the result of the matinée given on April 26th, in aid of the Fund.

H.R.H. the Duchess of Albany will open the new Convalescent Home of the National Hospital for the Paralysed and Epileptic (Albany Memorial), Queen Square, on Wednesday, June 16th. The home is at East Finchley, and has been provided for a class of patients inadmissible to every other convalescent institution in the kingdom. Only a small balance of the cost remains to be collected, and the board hope to be privileged to inform H.R.H. that the home is opened free of debt.

The Duke and Duchess of Connaught last week opened a bazaar at the County and Borough Hall, Guildford, in aid of the Soldiers and Sailors Families' Association and the Victorian Convalescent Home for Surrey Women. In thanking the committee for their welcome, the Duke said he was going to Windsor on the following day, and should have much pleasure in telling the Queen how the capital of Surrey had come forward and associated her name with an institution which would do a great deal of good in the country.

The question of a disputed legacy in the will of the late Sir Francis Lycett of £500 "to the King's Cross Hospital" was settled by Mr. Justice Kekewich in the Chancery Division of the High Court last week in favour of the Great Northern Central Hospital. There were several other claimants for the bequest, the Corporation of Dundee affirming that their hospital was the only one bearing the name of "King's Cross Hospital"; while the Small-Pox Hospital which originally stood on the site of the King's Cross Railway Station and Charing Cross Hospital each put in a claim. To the Great Northern Hospital both the testator and his wife had made donations during their lifetime. Mr. Justice Kekewich decided that this hospital was meant, to which, and to the trustees of the will and to the Attorney-General, he gave costs.

The minor branch of the Civil Service arranged a concert in aid of the Prince of Wales's Hospital Fund on the 22nd ult., at the Imperial Institute. A large number of influential persons patronised the entertainment.

A sum of £300, being one-half of a promised donation of £600, has been received from "Two Ladies" towards defraying the cost of the new cancer ward now being erected at Friedenheim, the Home of Peace for the Dying, Swiss Cottage, N.W.

Tourist arrangements, in connection with the forthcoming International Medical Congress at Moscow in August, are already being made; and Messrs. Cook and Dr. Lunn have already issued programmes of interesting tours to be carried out by visitors to the congress.

The After Care Association for poor persons discharged recovered from asylums for the insane is doing a useful work. The boarding out of suitable convalescents in cottage homes in the country has been found to work satisfactorily. Suitable homes are not easy to find, and the association is very glad to hear of those willing to render them assistance in this direction.

We are asked to state that the Bishop of Stepney has consented to preside at the annual meeting of the Hospital for Epilepsy and Paralysis, Regent's Park, which will be held at three o'clock on Wednesday, May 19th, at the hospital. Mr. T. Bryant, F.R.C.S., consulting surgeon to the hospital, and others have promised to be present.

Several Scottish charities have benefited largely by the will of the late Miss Brown, of Waterhaughs, Aryshire. Amongst other legacies £21,000 is bequeathed to the Home for Incurables at Kirkintillock, and a similar sum to the Edinburgh Association for Incurables. Twenty thousand pounds is left to a Fund for the Relief of Indigent Gentlewomen, and bequests of £5,000 apiece to the Edinburgh and Glasgow Infirmaries. The sum total to be distributed in this way amounts to £100,000.

At the last meeting of the Metropolitan Provident Medical Association the receipt of a handsome donation of £1,000 from the Baroness de Hirsch, towards the formation of new branches, was announced. The association has nineteen branches. An interesting experiment has been made during the year. In view of the ever increasing number of applications for chest hospital letters the Hospital Saturday Fund made a special grant to the Association, in return for which they undertook to sift the cases. The applicants are referred to one or other of the medical officers, who in return for a small fee states whether in his opinion the case is one for hospital or whether it is more suitable for home treatment. If the latter is found to be the case the patient receives a letter entitling him to a fortnight's treatment, which carries a fee of 3s. 6d. The fee may appear small but the cases are always of a slight nature, the more serious being sent to hospital. Mr. Acland, in his annual address to the Hospital Saturday Fund, reported that of the 1,101 cases so dealt with 51 per cent. were never heard of again; of the remainder, 313 were found suitable for chest hospitals, and letters were accordingly issued, 20 were sent to general hospitals, and 212 to the Metropolitan Provident Dispensaries.

